# Exploratory Spatial Lipidomic Profiling Reveals Regional Metabolic Heterogeneity in a Canine Mixed Mammary Carcinoma

**DOI:** 10.3390/vetsci13070689

**Published:** 2026-07-15

**Authors:** Mônica Duarte da Silva, Christina Ramires Ferreira, Hianka Jasmyne Costa de Carvalho, Sandra Maria Barbalho, Rodrigo da Silva Nunes Barreto, Rosa Direito, Maria Angélica Miglino

**Affiliations:** 1 Graduate Program in Anatomy of Domestic and Wild Animals, Faculty of Veterinary Medicine and Animal Science, University of São Paulo (FMVZ/USP), São Paulo 05508-270, SP, Brazil; monicasilva@usp.br; 2 Department of Chemistry and Biochemistry, The Ohio State University, Columbus, OH 43210, USA; decarvalho.8@osu.edu; 3 Bindley Bioscience Center, Purdue University, West Lafayette, IN 47907, USA; cferrei@purdue.edu; 4 Graduate Program in Structural and Functional Interactions in Rehabilitation, School of Medicine, Universidade de Marília (UNIMAR), Marília 17525-902, SP, Brazil; smbarbalho@gmail.com; 5 Laboratory for Systematic Investigations of Diseases, Department of Biochemistry and Pharmacology, School of Medicine, Universidade de Marília (UNIMAR), Marília 17525-902, SP, Brazil; 6 RB Laboratory, College of Agricultural and Veterinary Sciences, São Paulo State University (UNESP), Jaboticabal 17525-902, SP, Brazil; rodrigo.barreto@unesp.br; 7 Laboratory of Systems Integration Pharmacology, Clinical and Regulatory Science, Research Institute for Medicines, Universidade de Lisboa (iMed.ULisboa), 1649-003 Lisbon, Portugal; rdireito@ff.ulisboa.pt; 8 Graduate Program in Animal Health, Production and Environment, School of Veterinary Medicine, Universidade de Marília (UNIMAR), Marília 17525-902, SP, Brazil; 9 Department of Animal Anatomy, School of Veterinary Medicine, Universidade de Marília (UNIMAR), Marília 17525-902, SP, Brazil

**Keywords:** canine mammary tumors, lipid profiling, comparative oncology, spatial lipidomics, omics technology, intratumoral heterogeneity

## Abstract

Canine mixed-type mammary carcinomas (CMTs) exhibit remarkable histological and structural heterogeneity, sharing some biological similarities with human breast cancer and supporting their relevance as models in comparative oncology. These tumors frequently contain regions of malignant epithelial cells, areas of mesenchymal differentiation, and ossified tissue, reflecting their complex histological organization. In this exploratory pilot study, we performed MRM-based spatial lipidomic analysis of distinct tumor regions; namely the margin, middle (epithelial/myoepithelial-rich), and central ossified areas. Our region-resolved lipidomic analysis revealed distinct lipidomic signatures across regions, with significant alterations in phosphatidylcholines, phosphatidylethanolamines, sphingomyelins, triacylglycerides, and free fatty acids distribution. The central ossified area showed enrichment in structural lipids and ceramides, while the tumor margin demonstrated enrichment of ether lipids, which may reflect differences in tissue composition and local metabolic environments. By characterizing region-specific lipid profiles, this study provides insights into metabolic heterogeneity within a canine mixed mammary carcinoma. These findings highlight the potential of spatial lipidomics for investigating intratumoral metabolic heterogeneity and provide preliminary data to support future studies in comparative oncology involving canine and human mammary tumors.

## Simple Summary

Canine mammary cancer is one of the most common cancers affecting female dogs and shares many similarities with human breast cancer. Some canine mammary tumors exhibit complex histological organization, containing different tissue components, including epithelial, myoepithelial, mesenchymal, and bone-like areas. Understanding how these different regions vary may help explain regional differences in tumor biology. In this study, we examined lipid profiles and lipid-related molecules in three distinct areas of a canine mammary tumor: the outer edge, the middle cancerous region, and the central bone-like region. We found that each area had a unique chemical profile, showing that different parts of the same tumor exhibit distinct lipidomic signatures and metabolic characteristics. The middle region showed a unique lipid profile, while the central region exhibited lipid alterations that may reflect differences in tissue composition and local metabolic states. The tumor edge contained specific lipid species previously reported to be associated with cancer-related metabolic processes. These findings reveal important differences within the tumor that could not be detected by studying it as a single mass. Although based on an exploratory single-case analysis, these findings provide preliminary evidence supporting future investigations of regional lipid metabolism in canine mammary tumors and their potential relevance for comparative oncology.

## 1. Introduction

Canine mammary tumors (CMTs) represent a major health concern in female dogs, with high mortality rates reported. Due to notable anatomical, histopathological, and molecular similarities with human breast cancer, the canine species serves as a valuable spontaneous model for studying mammary carcinogenesis. In both species, tumors are classified based on glandular architecture, cellular pleomorphism, mitotic activity, and the degree of invasiveness [1]. Epidemiological parallels include similar sex and age distributions, and spatial cluster analyses have demonstrated overlapping geographic hotspots for mammary cancer in dogs and humans [2]. Additionally, key prognostic factors such as tumor size, histological subtype, stage, lymph node involvement, distant metastasis, and hormone receptor status are comparable between species [3]. These features support the use of CMTs in the investigation of disease progression, therapeutic strategies, and the identification of clinically relevant biomarkers [4,5].

Among human breast cancers, metaplastic breast carcinoma (MpBC) represents a rare and aggressive subtype characterized by the presence of epithelial and mesenchymal components, frequently exhibiting triple-negative molecular features and limited therapeutic options. In contrast, mixed mammary tumors are relatively common in female dogs and may display complex histological differentiation, including cartilage and osseous metaplasia. Although these tumors are not identical entities, their shared morphological complexity and the presence of mesenchymal components highlight the potential value of canine mammary tumors as comparative models for investigating mechanisms associated with tumor heterogeneity.

Among CMTs, mixed-type carcinomas are particularly heterogeneous, combining malignant epithelial elements with areas of mesenchymal differentiation, such as cartilage and bone [1]. This structural complexity suggests that these tumors may originate from pluripotent tumor cells and may reflect a high degree of tumor plasticity, rather than being directly linked to epithelial–mesenchymal transition (EMT), a process that has been proposed in the literature to be associated with increase invasiveness and poor prognosis in mammary tumors [3]. However, no direct molecular assessment of EMT was performed in the present study. Our research group has been actively investigating various biological and morphological aspects of CMTs, including the role of extracellular matrix components in tumor progression [6]. We have previously characterized the protein composition of mixed-type carcinomas in dogs [6], and, building on that foundation, we now aim to investigate the lipid profiles of distinct tumor regions, such as epithelial-dominant areas, chondroid tissue, and ossified regions, in order to explore spatial metabolic heterogeneity within the tumor.

Mass spectrometry (MS)-based lipidomics has become an important tool in cancer research, offering high sensitivity and specificity for identifying metabolic alterations associated with malignancy. Techniques such as liquid chromatography coupled with tandem mass spectrometry (LC-MS/MS), matrix-assisted laser desorption/ionization (MALDI), and desorption electrospray ionization (DESI) have been successfully applied to profile lipid species in tumors with spatial and molecular precision. In this context, multiple reaction monitoring (MRM) profiling has emerged as a particularly powerful quantitative MS technique, enabling targeted analysis of predefined lipid classes with high reproducibility and sensitivity [7]. Lipid profiling is especially valuable for surveying lipid biomarkers across distinct tumor regions, and for comparing lipid abundance associated with specific pathological features, such as regions with different cellular compositions or areas of mesenchymal differentiation. EMT-related transition zones or areas of mesenchymal metaplasia. EMT is associated with extensive metabolic reprogramming, including lipid profile, has been described in cancer literature and may contribute to tumor cell adaptation; however, such processes were not directly evaluated in this study [8,9].

Identifying such lipidomic signatures using MRM and other MS-based approaches may help generate hypotheses regarding tumor biology and metabolic heterogeneity. The main goal of this study is to provide a spatially resolved lipidomic characterization of mixed-type CMTs, thus contributing to comparative oncology and future investigations in both veterinary and human medicine. Integrated structural, molecular, and metabolomic analyses are essential to advancing diagnostic precision and improving therapeutic outcomes. However, given that this study is based on a single tumor case, all findings should be interpreted as exploratory and hypothesis generation rather than confirmatory.

## 2. Materials and Methods

### 2.1. Ethics Committee Approval

All experimental procedures conducted in this study were approved by the Ethics Committee on the Use of Animals at the School of Veterinary Medicine and Animal Science, University of São Paulo (FMVZ-USP), under protocol number 3966081220.

### 2.2. Biological Samples

A mammary tumor sample was collected from the inguinal gland of an adult female dog during a surgical excision mastectomy. After surgery, three biological replicates were collected from each of three distinct tumor regions: (R1) the tumor margin, (R2) the middle epithelial/myoepithelial-rich region, and (R3) the central region exhibiting osseous metaplasia. Fragments intended for lipid extraction were immediately stored at −80 °C. All samples analyzed in this study were derived from a single canine mammary tumor, and the three regions represented intra-tumoral biological replicates rather than independent animals. Therefore, statistical interpretations are limited to spatial heterogeneity within this individual case.

The histopathological analysis of the tumor was performed following the classification criteria established by Cassali et al. [1], and the tumor was diagnosed as a mixed-type mammary carcinoma. Detailed data obtained from histopathology and immunohistochemistry studies performed for this tumor characterization has been published by our group [6].

### 2.3. Lipid Extraction

To facilitate tissue homogenization and cell lysis, 100 μL of ultrapure water was added per 15 mg of canine tissue in a microtube containing the samples. The mixture was homogenized using a Precellys system at (Bertin Technologies, Montigny-le-Bretonneux, France) 5800 rpm until a homogeneous suspension was obtained. Lipid extraction was then performed following the Bligh and Dyer method with minor modifications [10]. Briefly, 200 μL of the homogenate was transferred to a new microtube, followed by the addition of 550 μL of methanol and 250 μL of chloroform. The solution was vortexed for 10 s to form a monophasic mixture and incubated at 4 °C for 15 min. To induce phase separation, 250 μL of ultrapure water and 250 μL of chloroform were added. The samples were centrifuged at 5000× *g* for 5 min, resulting in phase separation into: the organic phase (bottom layer) containing lipids, the aqueous phase (top layer), and a protein precipitate located at the interphase.

The organic phase was carefully collected and transferred to a new microtube. Solvent evaporation was performed using a SpeedVac concentrator (Thermo Fisher Scientific, Waltham, MA, USA) at room temperature. The dried lipid extracts were stored at −80 °C until mass spectrometry analysis. For MS analysis, the dried lipid extracts were resuspended in 200 µL of a solvent mixture composed of acetonitrile (ACN), methanol (MeOH), and ammonium acetate (NH_4_Ac) 300 mM in a ratio of 3:6.65:0.35 (*v*/*v*). After vortexing for 10 s, this solution served as the stock solution. Subsequently, 300 µL of the ACN:MeOH:NH_4_Ac solvent, containing the internal standard, was added to 1.5 µL of the stock solution.

Sample injections were performed using an automatic sampler (G1377A, Agilent Technologies, Santa Clara, CA, USA), delivering 8 µL per sample injection via flow injection analysis (FIA) directly into the ionization source of an Agilent 6410 triple quadrupole (QQQ) mass spectrometer (Agilent Technologies, Santa Clara, CA, USA). Each sample was analyzed in duplicate to ensure reproducibility of the monitored MRM transitions. The capillary pump operated at 150 bar with a flow rate of 20 µL/min. The capillary voltage was set between 3.5 and 5.0 kV, and the gas flow was maintained at 5.1 L/min at 300 °C.

### 2.4. Multiple Reaction Monitoring

MRM profiling analysis was conducted based on methods described by Reis et al. [11]. Initially, a list of MRM transitions was generated by pairing the mass-to-charge ratios (m/z) of molecular ions with their corresponding expected product ions for each lipid class or fatty acid residue.

MRM profiling was then performed on individual samples. In this study, three biological measurements (n = 3) correspond to three distinct tumor regions (R1–R3) obtained from a single canine mammary carcinoma, representing intra-tumoral spatial replicates rather than independent biological samples from different animals.

The final MRM transition list comprised 1586 entries, representing a consolidated set of 14,184 entries from the Lipid Maps database, where constitutional isomers were grouped into single entries to avoid redundancy in spectral interpretation.

This target approach enabled the monitoring of 10 distinct lipid subclasses, including phosphatidylcholines (PC), sphingomyelins (SM), phosphatidylethanolamines (PE), phosphatidylinositols (PI), phosphatidylglycerols (PG), phosphatidylserines (PS), triglycerides (TAGs), cholesterol esters, acylcarnitines, and free fatty acids (FFA).

### 2.5. Data Analysis and Statistics

The signal intensities of each MRM transition were initially processed through a quality filter, retaining only values greater than 30% of the intensity observed in the blank samples. After filtering, the intensities of each MRM transition were summed individually for each sample. Data were log-transformed prior to multivariate analysis and auto scaled (mean centered and unit variance scaled) where applicable.

To enable relative comparisons among the different transitions within each sample, the intensity of each MRM was normalized to the total sum of all transition intensities in the respective sample. This normalization allowed the intensities to be expressed as fractions of the total signal, facilitating proportional comparisons among transitions and reducing variability associated with differences in ionization efficiency and sample input.

For this exploratory single-case study, statistical comparisons among tumor regions were performed using one-way ANOVA to identify region-associated lipid differences. To correct for multiple testing associated with the analysis of multiple lipid features, *p*-values were adjusted using the Benjamini–Hochberg false discovery rate (FDR) correction method. Lipid features with FDR-adjusted *p*-values below the defined significance threshold were considered statistically significant.

Multivariate statistical analyses were performed using MetaboAnalyst 6.0 (www.metaboanalyst.ca, accessed on 25 April 2026). Principal component analysis (PCA) and hierarchical clustering analysis were performed as unsupervised approaches to evaluate global lipidomic patterns among tumor regions. Partial least squares discriminant analysis (PLS-DA) was performed as an exploratory supervised multivariate approach to identify lipid features contributing to the separation among tumor regions.

PLS-DA models’ performance was assessed using 5-fold cross-validation, with Q^2^ selected as the performance metric. A maximum of three components was considered during model optimization. Lipid features contributing most strongly to the discrimination among tumor regions were identified based on variable importance in projection (VIP) scores.

Given that all analyzed samples originated from a single canine mammary carcinoma, the biological replicates represented repeated sampling within each tumor region rather than independent biological samples from different animals. Therefore, all statistical analyses were interpreted as exploratory and intended to characterize spatial heterogeneity within this individual tumor.

**Figure 1:**
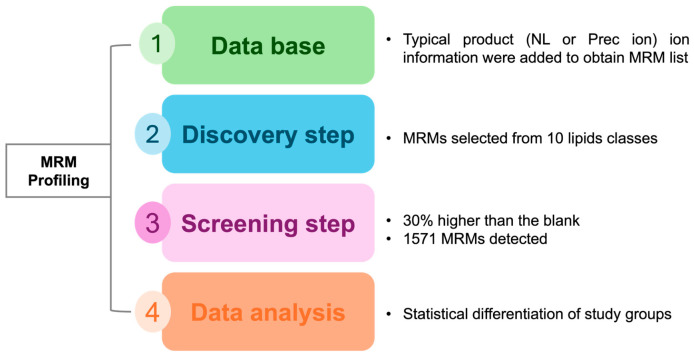
A schematic representation of the MRM profiling study workflow (created using BioRender).

An overview of the study design is provided in Figure 1.

## 3. Results

### 3.1. Principal Component Analysis (PCA)

**Figure 2:**
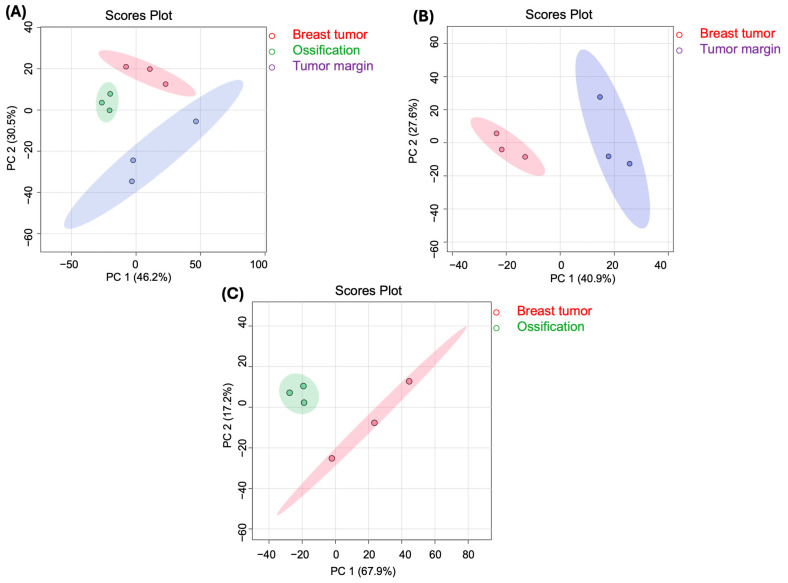
Principal Component Analysis (PCA) Results for the different regions of the tumor: (**A**) Region 1—tumor margin; (**B**) Region 2—middle region; and (**C**) Region 3—central region. The plots highlight the metabolic/lipidomic distinctions across spatial tumor areas.

PCA was conducted to investigate metabolic/lipidomic differences across distinct tumor regions. As shown in Figure 2, the score plot showed separation among the tumor regions, suggesting region-specific molecular profiles. The first two principal components accounted for 76.7% of the total variance (PC1: 46.2%, PC2: 30.5%), accounting for most of the observed variance of these components to capture the dataset’s variability. These findings reveal the presence of spatial metabolic heterogeneity within the tumor, which supports the presence of spatial metabolic heterogeneity across histologically distinct tumor regions.

### 3.2. Hierarchical Clustering Analysis and Heatmap Visualization

**Figure 3:**
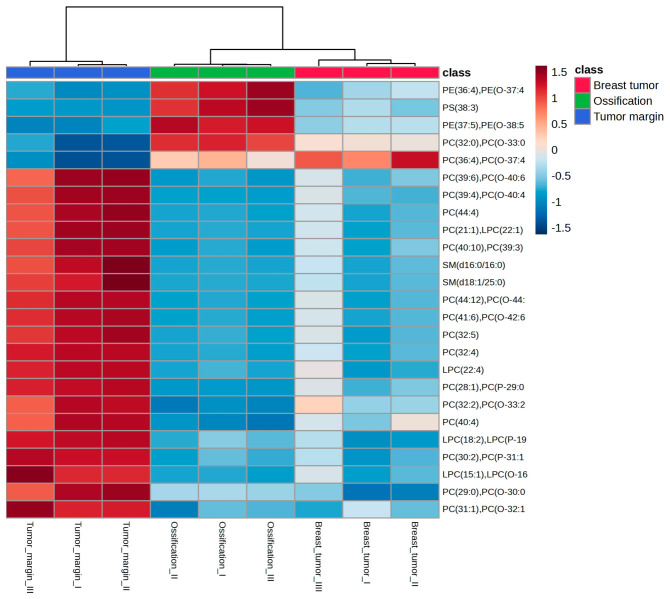
Heatmap showing the 25 of the 167 MRMs indicated as significant by ANOVA. Most lipids showing the greatest differences in relative abundance across three tumor regions of canine mixed-type mammary carcinoma: R1 (tumor margin), R2 (tumor middle), and R3 (ossification area). The color gradient represents relative lipid abundance, with red indicating higher expression and blue indicating lower expression. Distinct lipid expression patterns characterize each region, emphasizing the lipidomic heterogeneity within the tumor microenvironment. Lipid classes include phosphatidylethanolamines (PE), phosphatidylcholines (PC), sphingomyelins (SM), and lysophosphatidylcholines (LPC).

A heatmap analysis was performed to visualize the differential abundance of lipid species across the three defined tumor regions: (R1) the tumor margin, representing the peripheral area where the neoplastic process begins and residual healthy tissue may still be present; (R2) the middle and central regions of the breast tumor, characterized by malignant epithelial and myoepithelial components and corresponding to the tumor core; and (R3) the ossification area, associated with areas of dystrophic mineralization and altered microenvironmental conditions. As shown in Figure 3, unsupervised hierarchical clustering grouped the samples and lipids based on their relative intensities (Z-score scaled), revealing distinct lipidomic signatures associated with each region. The clustering of samples highlights the separation among R1 (blue), R2 (green), and R3 (red), indicating region-specific metabolic profiles.

Lipid species such as PE(36:4), PS(38:3), PE(37:5), and PC(32:0) displayed distinct region-specific expression patterns, showing markedly higher levels in the ossification region (R3), moderate levels at the tumor margin (R1), and lower relative abundance levels within the breast tumor core (R2). This gradient from low abundance at the invasive front to high accumulation at the tumor center suggests regional differences in lipid composition across the analyzed tumor regions progression and regional metabolic profiles. The heatmap emphasizes the metabolic heterogeneity of the tumor, especially between the invasive margin and the inner regions, and supports the presence of localized lipid heterogeneity, which may reflect differences in tissues composition and local microenvironmental.

### 3.3. Distinct Lipid Signatures Between the Middle (R2) and Central (R3) Regions of Canine Mixed-Type Mammary Tumor

**Figure 4:**
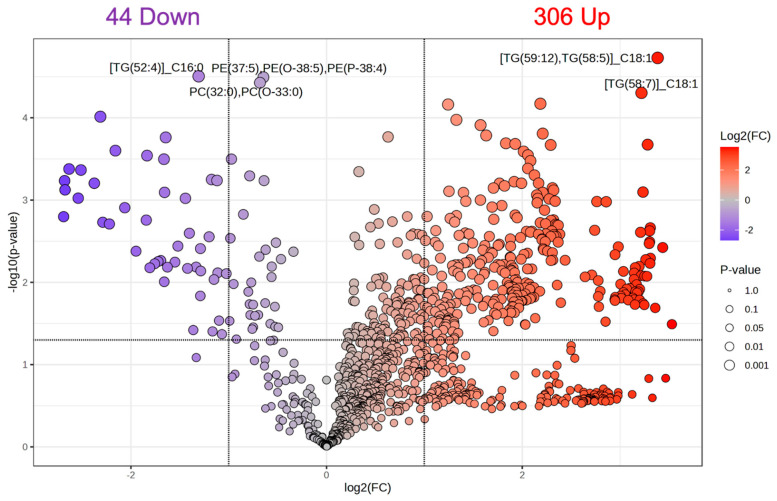
Volcano plot representing differentially regulated lipids between the middle and central regions of the canine mixed-type mammary carcinoma. The dashed horizontal line indicates the statistical significance threshold (*p* = 0.05), while the dashed vertical lines indicate the fold-change cutoff.

Lipidomic analysis comparing the middle (R2, breast tumor) and central (R3, ossification) regions of canine mixed-type mammary carcinoma revealed marked differences in lipid abundance (Figure 4). The middle region (R2), predominantly composed of malignant epithelial and myoepithelial cells, 45 showed lower relative abundance to the central region (R3), which is characterized by bone-forming tissue. Among the downregulated species were triacylglycerides such as TG(52:4), phosphatidylethanolamines including PE(37:5), PE(O-38:5), and PE(P-38:4). In contrast, the central region (R3) presented 330 higher relative abundance lipids, notably TG(59:12) and TG(58:5), the monounsaturated fatty acid oleic acid (C18:1), and ceramide Cer(d18:1/18:0). These distinct lipid expression profiles highlight distinct regional lipid profiles, potentially reflecting differences in tissue composition and local microenvironmental.

### 3.4. Distinct Lipid Signatures Between the Middle (R2) and Margin (R1) Regions of Canine Mixed-Type Mammary Tumor

**Figure 5:**
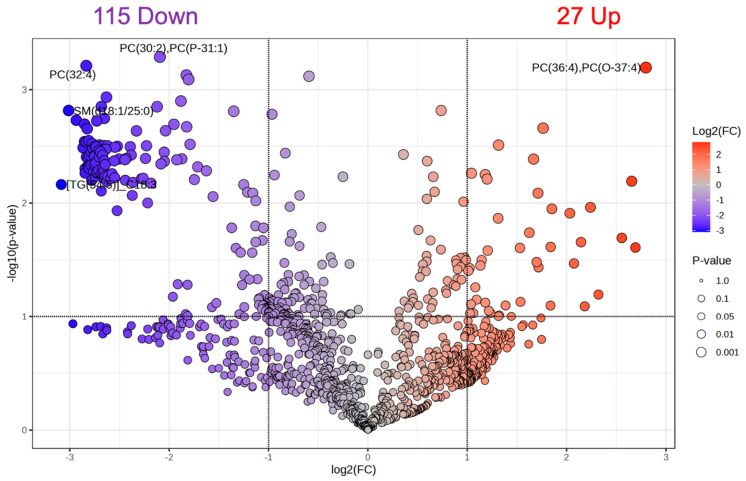
Volcano plot showing differentially regulated lipids between the middle region and tumor margin of canine mixed-type mammary carcinoma. The dashed horizontal line indicates the statistical significance threshold (*p* = 0.05), while the dashed vertical lines indicate the fold-change cutoff (|log2FC| = 1).

Comparative lipidomic profiling between the middle region (R2, breast tumor) and the tumor margin (R1) revealed significant differences in lipid regulation (Figure 5). A total of 183 lipids displayed differences in relative abundance (*p* < 0.05), with 141 lipids lower abundance and 42 higher abundance in R2 relative to R1. Notably, among the downregulated species were phosphatidylcholines such as PC(30:2), PC(P-31:1), and PC(32:4), the sphingomyelin SM(d18:1/25:0), and the triacylglyceride TG(54:5). Conversely, the upregulated lipids in R2 included phosphatidylcholines PC(36:4) and PC(O-37:4), the latter corresponding to an ether-linked phosphatidylcholine species. These findings underscore distinct lipid profile between the tumor core and its peripheral region, reflecting differences in the composition and accumulation of ether lipids.

### 3.5. Variable Importance in Projection Scores from Partial Least Squares Discriminant Analysis

**Figure 6:**
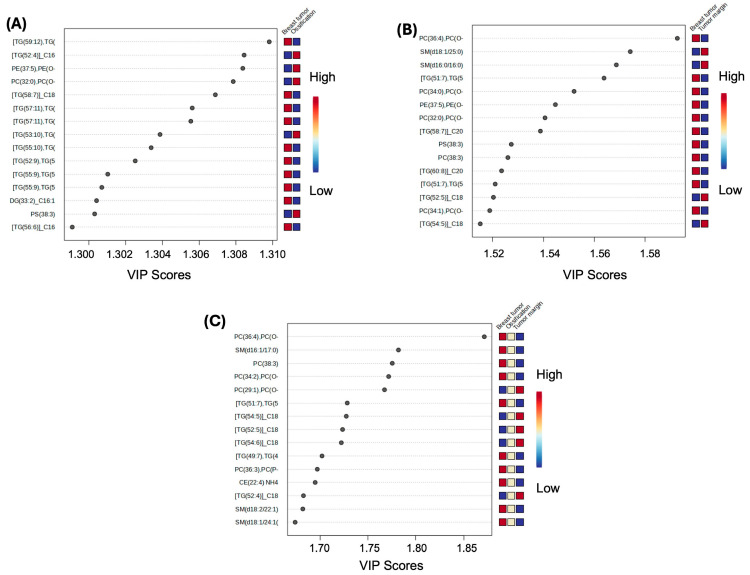
Variable Importance in Projection (VIP) score plots derived from exploratory-partial least squares discriminant analysis (PLS-DA) showing lipid features contributing to the separation among tumor regions. Comparisons include: (**A**) breast tumor (middle region, R2) vs. ossification region (central region, R3); (**B**) breast tumor (R2) vs. tumor margin (R1); and (**C**) multiclass analysis including all three tumor regions (R1, R2, and R3). Lipid features with the highest VIP scores represent variables contributing most strongly to the exploratory discrimination among regions. Heatmaps adjacent to each plot represent the relative abundance of selected lipid features across the analyzed tumor regions. The dashed line indicates the conventional VIP score threshold of 1.0, with variables above this threshold considered important contributors to group discrimination.

Three pairwise comparisons were performed to assess regional lipidomic differences within canine mixed-type mammary tumors. (A) Breast tumor (middle region, R2) vs. ossification (central region, R3): Triglycerides such as TG(59:12) and TG(52:4), phosphatidylethanolamines (e.g., PE(37:5)), and saturated fatty acids (e.g., C16:0) were among the highest contributing lipids differentiating the regions. (B) Breast tumor (R2) vs. tumor margin (R1): Key discriminatory lipids included phosphatidylcholines such as PC(36:4) and Sphingomyelin SM(d18:1/25:0), and TG(51:7), indicating distinct lipid profiles between the tumor core and periphery. (C) Multiclass analysis comparing all three regions (R1, R2, R3): Lipids such as PC(36:4), SM(d16:1/17:0), and PC(38:3) showed the highest VIP scores, indicating their greater contribution to the exploratory discrimination among tumor regions (Figure 6).

Heatmaps adjacent to each dot plot represent the relative abundance of each lipid species across the compared regions, with red indicating higher and blue lower expression levels.

## 4. Discussion

As demonstrated throughout this study, canine mixed-type mammary carcinoma offers a valuable comparative model for investigating metabolic alterations relevant to both veterinary and human oncology. By analyzing distinct histological regions within the tumor, represented by the margin, middle, and ossified central areas, we aimed to uncover spatially defined lipidomic signatures that reflect the metabolic heterogeneity of the tumor microenvironment. Our analysis revealed distinct lipidomic profiles across the analyzed tumor regions, consistent with spatial metabolic heterogeneity reported in previous studies [8,12,13]. The separation suggested by the univariate and unsupervised multivariate analyses, together with the differential patterns observed in the heatmaps, suggest the presence of regional differences in lipid composition within this individual tumor. These observations further support the utility of lipidomic profiling, which varies across histologically distinct tumor regions and may reflect differences in tissue composition and local metabolic states.

In this context, the elevated levels of phosphatidylcholines, such as PC 34:1 and PC 36:2, may reflect alterations in membrane lipid composition that have previously been associated with increased membrane biosynthesis in proliferating tumor cells. Additionally, the accumulation of sphingomyelins and ceramides, including SM 18:1/16:0 and Cer d18:1/24:0, has been associated with modulation of signaling pathways involved in apoptosis and inflammation in previous studies [14,15,16,17]. Conversely, the reduced abundance of polyunsaturated fatty acids (PUFAs), such as FFA 18:2 and FFA 20:4, may be consistent with altered lipid peroxidation pathways previously described in breast cancer [18]. Together, these observations demonstrate the potential of spatial lipidomic profiling to characterize spatial metabolic heterogeneity within canine mammary tumors and provide hypotheses for future validation studies.

The hierarchical heatmap displayed in Figure 3 reveals distinct lipidomic alterations among the tumor, tumor margin, and related ossification regions in the canine mixed-type mammary carcinoma. A prominent feature observed was the upregulation of phosphatidylglycerols alongside a consistent downregulation of sphingomyelins across most samples. These alterations may be associated with metabolic adaptations previously linked to oxidative stress [19,20]. Importantly, the observed clustering patterns represent exploratory observations derived from spatial regions within a single tumor and should not be interpreted as evidence of generalized tumor-associated lipid signatures.

Conversely, the reduction in sphingomyelins may be consistent with alterations in sphingomyelin-mediated signaling pathways, which have been described as regulators of apoptosis and tumor proliferation [15,20]. Based on previous reports, these alterations could potentially influence ceramide biosynthesis, since sphingomyelins are precursors in sphingolipid metabolic pathways. Ceramides are well-established antiproliferative mediators, promoting autophagy and apoptosis while inhibiting cancer cell growth and migration [14]. Together, these observations are consistent with previous reports suggesting that phosphatidylglycerols and sphingomyelins participate in biological pathways associated with metabolic plasticity and apoptosis regulation in canine mammary tumors [21,22].

Lipidomic profiling between the middle and central regions of the canine mixed-type mammary carcinoma revealed pronounced alterations in triacylglycerides and fatty acids that reflect the metabolic demands of the tumor microenvironment. The reduced relative abundance of triacyglycerides observed in the middle region is consistent with previous reports describing increased fatty acid utilization in rapidly proliferating tumors [12]. However, the present study did not directly assess fatty acid oxidation or metabolic flux, and therefore these interpretations should be considered hypothesis-generating.

Additionally, the downregulation of saturated fatty acids such as C16:0 (palmitate) and C18:1(0) may be consistent with alterations in lipid metabolism previously associated with tumor growth and apoptosis regulation [13,23]. Together, these findings suggest that the metabolic remodeling observed in the tumor’s middle region may reflect regional metabolic adaptations within the tumor microenvironment.

Lipid alterations further characterize the metabolic and structural remodeling occurring between the tumor and its margin in canine mixed-type mammary carcinoma. Notably, phosphatidylcholines (PCs) were less abundant in tumor regions than at the tumor margin, suggesting regional differences in membrane lipid composition. Previous studies have associated alterations in phosphatidylcholine metabolism with membrane remodeling and signaling pathways involved in tumor progression. Similarly, the downregulation of sphingolipids, particularly sphingomyelins, may be associated with alterations in sphingolipid metabolism, as previously reported in the literature.

Ether-linked phosphatidylcholines, particularly PC(O-37:4), have previously been associated with membrane remodeling and tumor aggressiveness in several cancer models [24,25,26,27]. Although similar alterations were observed in the present study, no functional analyses were performed to determine whether these lipids contribute to invasion, ferroptosis susceptibility, or metastatic behavior in canine mammary tumors. Therefore, these observations should be interpreted as potential hypotheses requiring further investigation.

The PLS-DA and VIP analyses were used as exploratory approaches to identify lipid features contributing to the separation among tumor regions. Although model performance was evaluated by cross-validation, the limited number of spatial regions derived from a single tumor restricts the robustness and generalizability of supervised multivariate models. Therefore, VIP-ranked lipid features should be considered candidate features associated with intratumoral metabolic variation rather than validated biomarkers. The present study has several limitations that should be acknowledged. The lipidomic analysis was performed on spatially distinct regions of a single canine mixed-type mammary carcinoma, limiting the generalizability of the findings. Moreover, the comparisons performed represent spatial differences within a single tumor rather than differences among independent biological samples. Although statistical analyses included multiple-testing correction using FDR adjustment, the exploratory nature and limited sample size require cautious interpretation of statistically significant lipid alterations. Furthermore, the study was designed to characterize regional lipidomic differences and did not include functional experiments or molecular analyses to directly investigate biological processes such as epithelial–mesenchymal transition, apoptosis, ferroptosis, or lipid metabolic pathways. Consequently, the proposed biological interpretations are based on previously published literature and should be considered hypothesis-generating. Validation in larger cohorts and complementary functional studies will be necessary to confirm these observations.

From a comparative oncology perspective, these findings provide preliminary evidence that spontaneous canine mixed-type mammary carcinomas may serve as valuable models for investigating spatial metabolic heterogeneity relevant to both veterinary and human breast cancer. However, larger studies integrating lipidomics with histopathological, molecular, and functional analyses will be necessary to determine whether similar regional metabolic signatures are conserved across species.

The present study provides an exploratory spatial characterization of lipidomic heterogeneity within a canine mixed-type mammary carcinoma. Although limited to a single case, the observed regional lipid differences provide preliminary evidence supporting further investigation of intratumoral metabolic heterogeneity. Future studies integrating lipidomics with histopathology, transcriptomics, and functional analyses will be important to determine the biological and clinical significance of these lipid alterations in both veterinary and comparative oncology.

## 5. Conclusions and Future Research Directions

The present exploratory study identified regional lipidomic differences across the tumor margin, middle, and central ossification regions of a canine mixed-type mammary carcinoma, providing preliminary evidence of spatial metabolic heterogeneity within this tumor. These findings reinforce the importance of region-specific analyses, as evaluating the tumor as a homogeneous entity may overlook biologically relevant regional lipid alterations. Although limited to a single case, the observed lipidomic differences likely reflect regional differences in tissue composition and local metabolic states across distinct histological regions, although confirmation in larger cohorts is still required. These exploratory findings provide a foundation for future studies investigating intratumoral lipid heterogeneity in larger cohorts and integrating lipidomics with histopathological, molecular, and functional analyses. Such approaches may contribute to a better understanding of mammary tumor biology in both veterinary oncology and comparative oncology.

## Data Availability

The original contributions presented in this study are included in the article. Further inquiries can be directed to the corresponding author.
